# Phosphatidylserine inside out: a possible underlying mechanism in the inflammation and coagulation abnormalities of COVID-19

**DOI:** 10.1186/s12964-020-00687-7

**Published:** 2020-12-27

**Authors:** Gustavo A. Argañaraz, Julys da Fonseca Palmeira, Enrique R. Argañaraz

**Affiliations:** Laboratory of Molecular Neurovirology, Faculty of Health Science, University of Brasília, Brasília, 70910-900 Brazil

**Keywords:** COVID-19 pathophysiology, DIC-COVID-19, ADAM17, Phosphatidylserine

## Abstract

The rapid ability of SARS-CoV-2 to spread among humans, along with the clinical complications of coronavirus disease 2019—COVID-19, have represented a significant challenge to the health management systems worldwide. The acute inflammation and coagulation abnormalities appear as the main causes for thousands of deaths worldwide. The intense inflammatory response could be involved with the formation of thrombi. For instance, the presence of uncleaved large multimers of von Willebrand (vWF), due to low ADAMTS13 activity in plasma could be explained by the inhibitory action of pro-inflammatory molecules such as IL-1β and C reactive protein. In addition, the damage to endothelial cells after viral infection and/or activation of endothelium by pro-inflammatory cytokines, such as IL-1β, IL-6, IFN-γ, IL-8, and TNF-α induces platelets and monocyte aggregation in the vascular wall and expression of tissue factor (TF). The TF expression may culminate in the formation of thrombi, and activation of cascade by the extrinsic pathway by association with factor VII. In this scenario, the phosphatidylserine—PtdSer exposure on the outer leaflet of the cell membrane as consequence of viral infection emerges as another possible underlying mechanism to acute immune inflammatory response and activation of coagulation cascade. The PtdSer exposure may be an important mechanism related to ADAM17—mediated ACE2, TNF-α, EGFR and IL-6R shedding, and the activation of TF on the surface of infected endothelial cells. In this review, we address the underlying mechanisms involved in the pathophysiology of inflammation and coagulation abnormalities. Moreover, we introduce key biochemical and pathophysiological concepts that support the possible participation of PtdSer exposure on the outer side of the SARS-CoV-2 infected cells membrane, in the pathophysiology of COVID-19.

**Video Abstract**

**Video Abstract**

## Background

Coronavirus disease 19 (COVID-19) has already caused hundreds of thousands of deaths and has become one of the humanity’s greatest health challenge in our time [1]. The acute inflammation and disseminated intravascular coagulation appear as the main causes of mortality worldwide [2–4]. Efforts to overcome this challenge have been limited by worldwide hospital capacity and a lack of knowledge about crucial aspects of the infection and effective treatment options. Faced with this, several therapeutic options have been studied and tested in order to find new strategies to limit/block viral entry and improve the main clinical complications related to the disease. Moreover, clinical interventions have emerged and brought important advances, such as reducing mortality and improving the prognosis of critically ill patients treated with anticoagulant/anti-inflammatory drugs [5]. However, the molecular bases underlying the main pathophysiological anomalies related to COVID-19 remain incompletely understood.

In this context, the phosphatidylserine—PtdSer exposure on the outer leaflet of the membrane emerges as a possible underlying phenomenon in the pathophysiology of COVID-19. Under physiological conditions, the PtdSer exposure on the outer leaflet of activated platelets provides a platform for the aggregation of various coagulation factors [6], is involved with red cell senescence, cell activation and/or death [7–10] and with inhibition of activation of inflammatory and autoimmune mechanisms [11, 12]. However, in pathophysiological conditions, the PtdSer exposure may have deleterious effect relate to coagulation activation [13] and with viral infections [14–16], increasing the infectivity and viral spread [17–21].

In this work, we introduce key biochemical and pathophysiological evidences regarding the main mechanisms involved in the genesis of coagulation abnormalities and, in particular, the involvement of endothelial cells damage and/or activation-mediated by virus infection and acute inflammatory response. In this scenario, we also discussed the possible participation of phosphatidylserine-PtdSer exposure on the outer leaflet of the membrane of SARS-CoV-2 infected cells as a possible underlying mechanism to acute immune inflammatory response and activation of coagulation cascade.

## SARS-CoV-2 infection and the renin-angiotensin system—RAS

The SARS-CoV-2 virus uses the surface spike (S) proteins for host cell attachment and infection. The precursor of S viral glycoprotein is cleaved into S1 and S2 polypeptides by furin, a host cell protease, in a polybasic site, RRAR^S, at the S1–S2 junction, which is absent in the SARS-CoV. This difference may be responsible for a more efficient cleavage of SARS-CoV-2 protein S, than that of the SARS-CoV, and, thus, explain the higher SARS-CoV-2 infectivity [22–24]. In addition, the polybasic sequence motif, RRAR, at C-terminal sequence of the S1, termed the 'C-end rule' (CendR) [22–24], can bind to the cell surface Neuropilin receptors (NRP1 and 2) [25–27]. The S1 and S2 proteins remain noncovalently linked and, after binding of SARS-S to the angiotensin-converting enzyme 2 (ACE2), the S2 protein is subsequently cleaved by a type II transmembrane serine protease (TTSP), TMPRSS2 [28]. Alternatively, another less important route of infection involves cathepsin L, a pH-dependent endo-lysosomal host cell protease, after the uptake of virions into target cell endosomes [29].

**Fig. 1:**
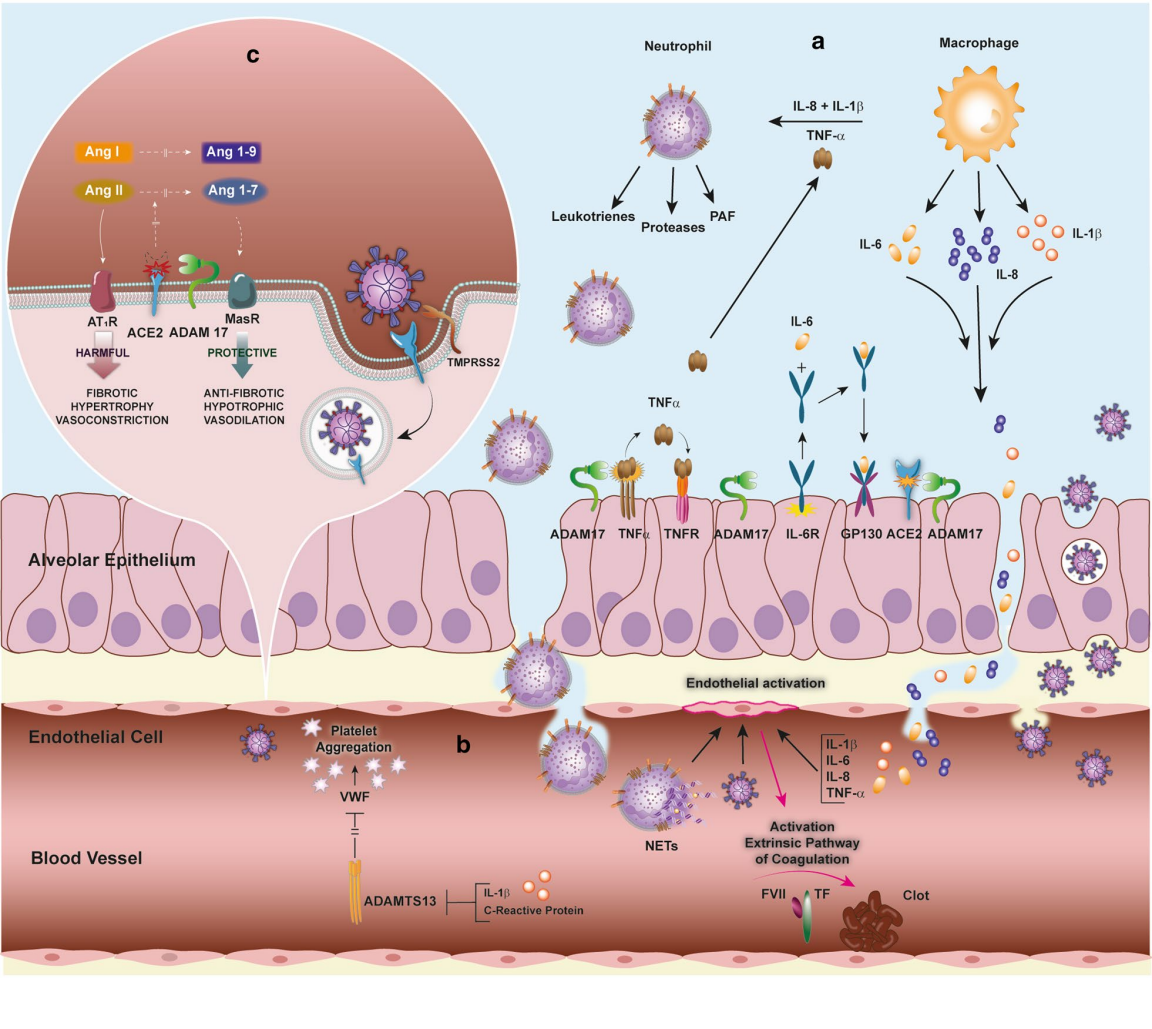
Molecular mechanisms involved in the acute inflammation and coagulation abnormalities of COVID-19. **a** The SARS-CoV-2 infection triggers an inflammatory cellular infiltrate in the alveolar lumen releases toxic molecules by macrophages and neutrophils, such as IL-1β, IL-8, IL-6 and TNF-α. The cytokines storm lead to diffuse alveolar damage, pulmonary oedema. **b** The damage and /or activation of blood vessels endothelium by viral infection and pro-inflammatory cytokines respectively induce platelet and monocyte aggregation in the vascular wall. These events are accompanied by increased expression of the tissue factor (TF) leading to activation of extrinsic pathway coagulation cascade culminating with the thrombi formation. Moreover, the thrombotic microangiopathy, may also be consequence of uncleaved large multimers of VWF, due to a decrease in the plasma levels of ADAMTS13. **c** Finally, as consequence of blood vessels endothelium viral infection, ACE2 is internalized, and sheddase activity of ADAM17 is increased. The ACE2 downmodulation and TNF-α and IL-6R release exacerbates the imbalance of RAS, leading to increase inflammation. The virus-mediated PtdSer exposure on the outer leaflet of the cell membrane emerge as an underlying mechanism to activate TF and extrinsic pathway coagulation cascade and inflammation (not shown)

ACE2 is a negative regulator of the renin-angiotensin system (RAS), converting angiotensin (Ang) I and Ang II into Ang 1–9 and Ang 1–7, respectively [30] and, thus, protecting the cardiovascular system against systemic hypertension, myocardial infarction and diabetic cardiovascular complications [31, 32]. As a consequence of viral infection, ACE2 is internalized along with viral particles into endosomes, decreasing tissue ACE2 expression [33, 34]. Among the main consequences of RAS imbalance are those related to vasoconstriction, hypertrophy, fibrosis and acceleration the thrombin generation pathway, as well as inflammation in COVID-19 patients [35, 36]. ACE2 downmodulation is intensified by the cleavage of cellular proteases, the disintegrin and metalloproteinase domain 17 (ADAM-17) and TMPRSS2, which enhance the imbalance in the RAS and leads to increased Ang II plasma levels in COVID-19 patients [37, 38]. The TMPRSS2-mediated ACE2 cytoplasmic tail cleavage may lead to an increase in viral uptake through the cathepsin L-dependent pathway [38]. Interestingly, the ACE2 cytoplasmic tail, as well as ADAM17 expression have been found to be necessary to viral infection; however, the underlying mechanism by which ACE2 tail-mediated ADAM17 activation facilitates viral entry is still unclear. In particular, the internalization of ACE2 would trigger a harmful positive feedback pathway, since the decrease in ACE2 expression results in an increase in Ang II, which in turn leads to an excessive activation of AT1Rs, increasing ADAM17 activity and the shedding of ACE2, and, thus depleting the tissue of ACE2 even more [39, 40]. Moreover, the up-regulation of ADAM17 activity may be triggered by increasing of IL-β levels, a pro-inflammatory cytokine induced by SARS-CoV-2 infection [41] (Fig. 1c). Finally, the increased ADAM-17 catalytic activity to remove the membrane proteins ectodomains (sheddase activity) also leads to the cleavage of TNF-α and interleukin-6 receptor (IL-6R), as well as other pro-inflammatory molecules, reinforcing the inflammatory process [31, 42].

Several findings support the pathophysiological relevance of increased ADAM17-mediated ACE2 tissue degradation in both COVID-19’s comorbidities and SARS-CoV-2 infection: (1) increased plasma levels of ACE2 soluble form (sACE2) with age in men [43, 44]; (2) higher expression levels of sACE2 in men with heart failure than woman, as consequence of increased ADAM17 activity [45]; and (3) higher ACE2 level expression and up-regulated activity of ADAM17 in patients with chronic pulmonary inflammation [46], chronic obstructive pulmonary disease (COPD) [43, 47], diabetes [48, 49] and renal diseases [50].

The relevance of increased ADAM17-mediated ACE2 tissue degradation on cardiovascular diseases has been highlighted by Sama et al. [45]. In that study, conducted in patients with heart failure, the ACE2 plasma levels were higher in men than in women with heart disease. The higher plasma levels of ACE2 have been interpreted as a consequence of higher ADAM17 activity, and as molecular marker of a poor prognosis [40]. In support of that interpretation, elevated levels of TNFα have been found in heart diseases such as myocarditis and correlated negatively with left ventricular systolic function in patients [51].

## Pathophysology of COVID-19

### Acute respiratory distress syndrome

COVID-19 is associated with a high transmissibility rate when compared to other SARS-CoV diseases [52]. Symptomatic patients may have mild to severe clinical respiratory manifestations, or very severe sequelae such as those resulting from acute respiratory distress syndrome (ARDS). The progression of some patients to ARDS may be related to a genetic susceptibility that promotes an imbalance between pro-inflammatory and anti-inflammatory mediators [2], leading to an exacerbated immune-inflammatory response. This clinical syndrome is characterized by the following features: the acute establishment of dyspnea that progresses to severe respiratory failure; a reduction in oxygen partial pressure (hypoxemia); bilateral pulmonary infiltrates; and clinical absence of left heart failure. SARS-CoV-2 infection causes virus-related epithelial pyroptosis in type I and II pneumocytes and activation of macrophages in the pulmonary alveoli [53]. Activated macrophages are the main source of pro-inflammatory cytokines such as IL-1β, IL-6, IFN-γ, IL-8, and TNF-α [54, 55]. These cytokines activate the acute inflammatory response due to increased endothelial permeability and a chemotactic effect on neutrophils, monocytes, and cytotoxic T lymphocytes. These inflammatory cellular infiltrates in the alveolar lumen subsequently release toxic molecules, leading to diffuse alveolar damage, pulmonary edema, and fibrin deposition (hyaline membrane) into the alveolar space [4] (Fig. 1a).

### Inflammation and coagulation abnormalities

The unfavorable evolution of ARDS patients is connected with multiple organ failure, which in turn is strongly associated with coagulation abnormalities such as thrombotic events; microvascular platelet-rich thrombotic deposition in different organs [3, 4, 56] have been observed during early stages of COVID-19, while disseminated intravascular coagulation (DIC) has been observed in later stages [57]. The latest anatomopathological data are related to thrombotic microangiopathy. The predisposition to the arterial and venous thrombi formation in patients with COVID-19 may be related to two main non-exclusive and related mechanisms: the intense inflammatory response and cell injury by virus infection.

The intense inflammatory response and thrombi formation are interrelated via positive feedback [58]. In early stages of the inflammatory response, the thrombotic phenomena could be facilitated by two different ways. The first may involve passage of inflammatory mediators to the circulation, such as the pro-inflammatory cytokines TNF-α, IL-1β and IL-6, generated at the pulmonary tissue [54]. The activation of endothelium by these pro-inflammatory cytokines facilitate platelet and monocyte aggregation, as well as the expression of a glycoprotein known as a tissue factor (TF) in the vascular wall. The increased expression of TF, both in endothelial cells and monocytes, may activate the coagulation cascade culminating in the formation of thrombi [58–60] by binding to factor VII, forming the TF/FVIIa complex (Fig. 1c). A study conducted by Ethan et al. [61] analyzing transcriptomic data sets of SARS-CoV-2 human infected bronchial epithelial cells found significantly increased F3 gene expression encoding TF protein [61]. These findings strongly suggest that hyper-activation of the extrinsic blood coagulation cascade in patients with COVID-19 may be a critical mechanism for the development of coagulopathies. In addition, the increased IL-6 levels were correlated with increased fibrinogen, linking inflammation to abnormalities of coagulation [62]. These phenomena might explain the laboratory data demonstrating high levels of D-dimer, mild decrease platelet count, increase plasma fibrinogen levels, low levels of anti-thrombin, and prothrombin time extension (PT), as well as the clinical manifestations of DIC in later stages in patients with COVID-19 [63]. The other mechanism involves the IL-1β and C reactive protein-mediated large von Willebrand factor multimers (vWF) accumulation in plasma. This mechanism may be due to decreased activity of a disintegrin or metalloprotease with thrombospondin type 1 repeats 13—ADAMTS13. The ADMTS13 downmodulation could be explained by the inhibitory effect of pro-inflammatory molecules such as IL-1β and C reactive protein, as shown by Chen and colleagues in patients with lymphoblastic leukemia [64]. Indeed, several studies have shown that plasma of COVID-19 patients with microangiopathy contains low ADAMTS13 activity and increased vWF and factor VII levels [65, 66]. However, in a recent study conducted by Escher and colleagues, no role of ADAMTS13 in the pathogenesis of COVID-19 coagulopathy was observed [67].

Regarding injury or endothelial cells dysfunction as a result of virus infection, three main pathophysiological consequences are possible. One consequence is the aggregation of platelets, leading to an imbalance between pro- and anti-anticoagulant agents. In this sense, Zhang et al. [68] showed that SARS-CoV2 virus directly activates platelets through the spike/ACE2 interactions, potentiating their prothrombotic function and inflammatory response. These events correlate with histopathological findings in pulmonary vessels of COVID-19 patients, such as the high incidence of alveolar capillary microthrombi, pulmonary thromboembolism, and other vascular occlusive events (e.g. ischemic limbs, strokes) [69–71]. A second consequence is an increase in the level of vWF and infiltration of active neutrophils and macrophages (endothelialitis), and the formation of neuthrophil extracellular traps (NETs) [72, 73]. The NETs can increase damage to the endothelium, and consequently activate both extrinsic and intrinsic pathways [74]; this could cause development of a prothrombotic state that could be facilitated by hypoxia-inducible factor (HIF) after lung injury [75]. One final possible consequence of viral infection is the internalization of ACE2 receptor from the surface of epithelial cells in the pulmonary alveoli, AT1Rs activation [76], and ADAM-17 and TMPRSS2 cleavage. The ACE2 downmodulation causes an imbalance of the RAS system, leading to an increase in Ang II plasma levels and facilitating thrombosis via a thrombin-dependent pathway (Fig. 1b) [35].

## PtdSer translocation and viral infections

PtdSer is one of the main acidic and the most abundant negatively charged phospholipids in mammalian cell membranes [77, 78]. PtdSer is asymmetrically distributed between the inner and outer leaflets of the lipid bilayer, and it is translocated in response to membrane perturbation, cell damage or intracellular signals [79]. Transient PtdSer exposure has been reported in conditions such as PKC activation [80] and cytosolic Ca^2+^ elevation [81], showing that diverse stimulus that can lead to exposure of PtdSer to the outer membrane leaflet. PtdSer translocation can be triggered basically by two mechanisms: elevated Ca^2+^ ionophores or apoptosis [82]. Both processes are accompanied by the activation of “scramblases”, proteins that expose PtdSer by translocating phospholipids between the inner and outer leaflets of the plasma membrane to the cells surface [83]. During apoptosis, caspase-dependent Xkr8 (Xkr8) activation leads to irreversible “flippase” inactivation. In contrast, in response to cytoplasmic Ca^2+^ elevation, the transmembrane 16F lipid-scramblase protein (TMEM16F/anoctamin 6) is activated and translocates PtdSer to the outer side of the cell membrane [83]. In addition, TMEM16F can act as a regulator of Ca^2+^ activated membrane traffic [84] or promote plasma membrane repair after pore formation [85]. PtdSer exposure acts as a signal for dead cell phagocytosis by macrophages, avoiding the activation of inflammatory and autoimmune mechanisms [11, 12]. Phagocytosis associated with PtdSer translocation could be an early event associated with viral infections, as described with influenza A virus [14], HIV-1 [15], and herpes simplex virus-1 [16]. Moreover, enveloped viruses like HIV-1, Ebola (EBOV), West Nile, Dengue and Zika viruses incorporate PtdSer to increase viral entry by binding to T-cell immunoglobulin (Ig) and mucin domain (TIM) proteins [17–21]. In a recent study, it was shown that HIV-1 activates TMEM16F “scramblase” to expose PtdSer on the outer leaflet of host cell membrane to increase viral infectivity [86]. Although this mechanism has not yet been addressed in coronavirus infection, PtdSer translocation by TMEM16F-scramblase activation cannot be ruled out and deserves further investigation.

## PtdSer and pathophysiology of COVID-19

### PtdSer exposure in pathophysiological conditions

Exposure of PtdSer on the outside of the cell membrane also occurs under certain biological conditions such as platelet activation, microvesicle shedding from cell surfaces, anoxia, red cell senescence, and cell activation or death [7–10]. Coagulation plays a critical role in hemostasis and in the innate immune response to infection in an effort to avoid the dissemination of microbes [87]. However, under some pathological conditions, the activation of coagulation could be deleterious. Indeed, in a mouse endotoxemia and bacterial sepsis model, caspase-11, a cytosolic lipopolysaccharide (LPS) receptor, enhanced the pro-coagulant activity of TF independent of cell death. The activation of caspase-11 increased calcium influx through the formation of gasdermin D (GSDMD) pores, which led to TMEM16F scramblase-mediated PtdSer exposure [13]. In addition, the formation of GSDMD pores by K^+^ efflux led to activation of the NOD-like receptor (NLR) family pyrin domain-containing 3 (NLP3) inflammasome and increases in the release of IL-1β. The IL-1β and IL-1α levels in septic patients were significantly correlated with the PtdSer exposure on the outer side of the leukocyte membrane and with the DIC score. Caspase 11 deletion significantly inhibited LPS-induced thrombin and thrombin-antithrombin complex (TAT) generation, D-dimer, platelet aggregation, and plasminogen activator inhibitor type 1 (PAI-1) in plasma [13]. On the outer side of the membrane, PtdSer is able to positively modulate TF activation and initiate the coagulation cascade [88]. The transcriptional upregulation of TF in the NHBEs support the possibility of PtdSer-mediated TF activation in infected endothelial cells [61]. Moreover, under physiological conditions, Ca^2+^-mediated PtdSer exposure on the outer leaflet of activated platelets provides a platform for the aggregation of various coagulation factors [6]. In Scott syndrome, a bleeding disorder, the lack of procoagulant activity in activated platelets is related to a mutation in the TMEM16F “scramblase” protein [89].

### PtdSer exposure and COVID-19 inflammatory response

**Fig. 2:**
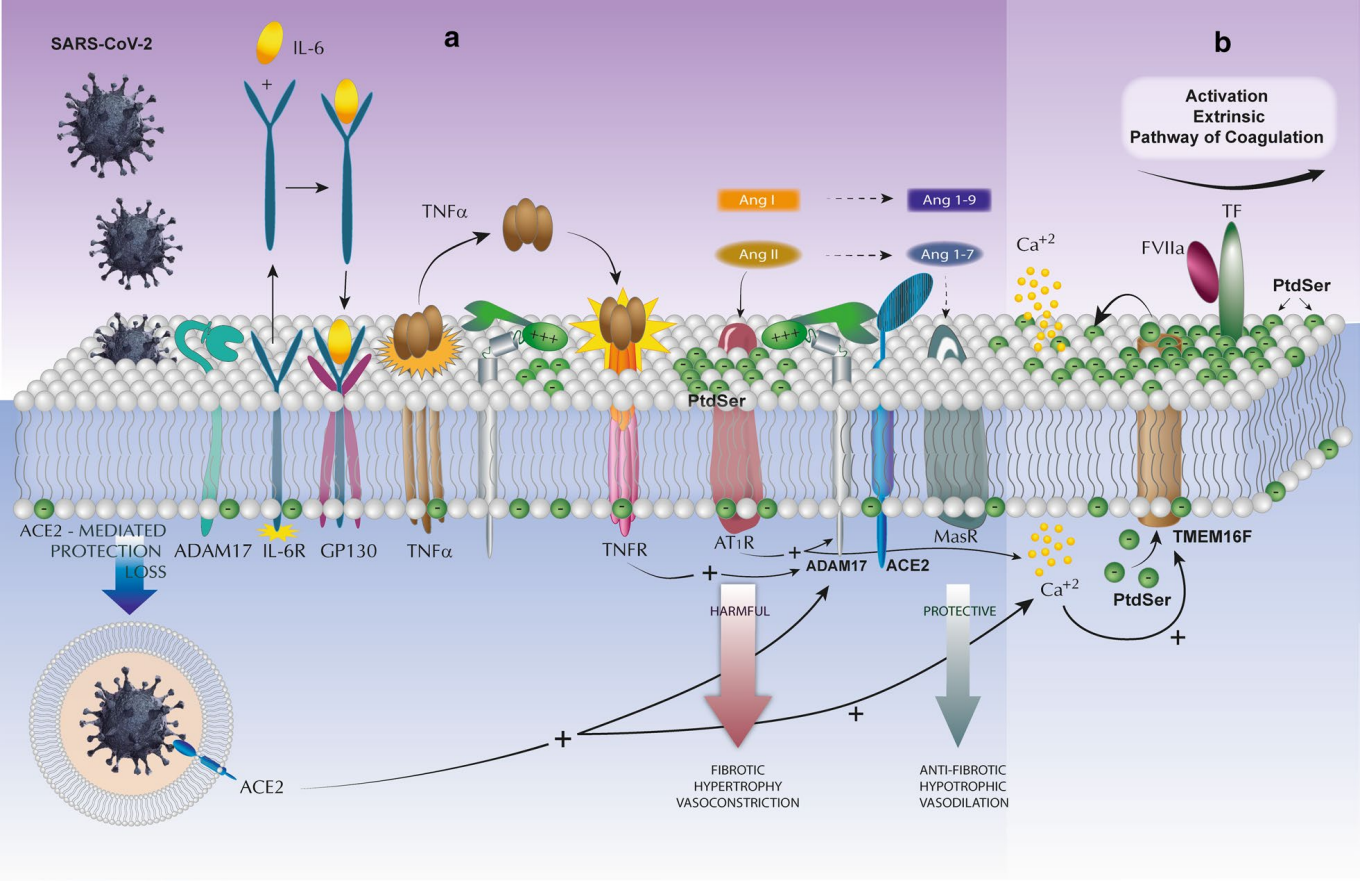
Model of ADAM17 and extrinsic pathway of coagulation cascade activation by PtdSer externalization. **a** As a consequence of SARS-CoV-2 infection, ACE2 is internalized, increasing the Ang II/AT1R-axis. Both stimuli, ACE2 down-modulation and the high Ang II level trigger intracellular signals that culminate in Ca^2+^ influx, and TMEM16F-scramblase activation, leading to PtdSer externalization to the outer cell membrane. The interaction between the ADAM17 cationic conserved sequence domain with the negatively charged PtdSer head group on the outer leaf membrane, bring the protease in the right position for ACE2 processing, with detrimental effects. Additionally, the ADAM17 “shedasse” activity also release of proinflamatory cytokine TNF-α and IL-6R increasing inflammatory process**. b** Finally, the PdSer exposure on the outer membrane may also activate the extrinsic pathway of coagulation cascade by enhancing the activation of tissue factor, being able to contribute to disseminated intravascular coagulation—DIC observed in COVID-19 patients

The molecular mechanisms by which ADAM17 is regulated have not yet been fully elucidated, but would depend on the stimulus and the cell type. For instance, the release of the soluble active TNFα form and of the epidermal growth factor receptor (EGFR) by ADAM17 depends on iRhom2 protein, an inactive member of the rhomboid family, and would be essential for maturation and substrate specificity of ADAM17 [90]. The ADAM17 activity can be induced by other molecular stimuli including PKC activators, purine 2 (P2) receptor agonists, fibroblast growth factor 7 (FGF7), Ca^2+^ ionophores, and membrane perturbations [91, 92]. ADAM17 can also be activated in response to infection by pathogens through Toll-like receptor activation [93, 94]. PtdSer on the outer side of the cell membrane also plays an important role as a docking site for enzymes such as Src kinase and protein kinase C (PKC), establishing protein-lipid interactions sustained by electrostatic forces between the negatively charged phospholipid head group and cationic amino acid clusters [77]. Indeed, the PtdSer translocation was described as important requirement for ADAM17 to exert its “sheddase” activity [82] (Fig. 2a). Elliott et al. [95] showed that rapid PtdSer translocation was correlated with release of the ADAM17 substrate L-selectin after stimulation with purinergic receptor (P2) receptor agonists, independent of any apoptotic events. In this context, Sommner et al. [82] propose a model of ADAM17 membrane sheddase activity and PtdSer exposure. In this model, the interaction of PtdSer and ADAM17 brings the protease into position for substrate processing. A recent work has shown that exposure of PtdSer is required for activation of ADAM10 sheddase activity [96]. The same PtdSer cationic binding motif as that identified in ADAM17 has also been identified [82, 97]. However, as opposed to the ADAM17 shedadase activation mechanism, Anoctamin-6, a Ca^2+^-activated ion channel that also functions as a scramblase, would be responsible for shuttling PtdSer from the internal to the outer membrane leaflet and ADAM10 activation [96]. Taking into account that the ADAM17 sheddase activity can remove the membrane protein ectodomains, the PtdSer-mediated ADAM17 activity may be the underlying mechanism for the cleavage of ACE2, TNFα, IL6R, EGFR, and other pro-inflammatory molecules; these are critical components in the inflammatory process during SARS-CoV-2 infection.

### PtdSer exposure and COVD-19 coagulopathy

The coagulation abnormalities observed in COVID-19 patients may be linked to changes in the plasma homeostasis of membrane phospholipids. The extracellular PtdSer exposure could activate the body’s inflammatory and coagulation cascades. In support of this hypothesis, Zang et.al demonstrated the presence of antiphospholipid antibodies targeting phospholipid proteins (Anticardiolipin IgA, anti-β2 glycoprotein I IgA and IgG), characteristic of antiphospholipid syndrome (APS), in three patients with admitted to the ICU with COVID-19 and coagulopathy [98]. Interestingly, Zigon et al. [99] showed a strong correlation between the antiprotrombin and PtdSer/Protrombin complex antibodies in patients with clinical manifestations of APS, such as arterial and venous thromboses and obstetric complications. These data raise the possibility of phospholipid involvement in the pathophysiology of coagulopathies in patients with COVID-19, and the use of these serological markers of severe coagulopathy in COVID-19 patients. Alternatively, PtdSer translocation is related to elimination of senescent and damaged cells via binding to membrane receptors on macrophages, avoiding spillage of cellular contents and inflammation [100, 101]. However, during sepsis or inflammatory events like SARS-CoV-2 infection, PtdSer exposure could be up-regulated on cell surfaces throughout the body, including endothelial cells, platelets, erythrocytes, neutrophils, lymphocytes, and extra cellular microparticles. The possible underlying mechanism of these events could be related to the super-activation of TMEM16F scramblase along with PtdSer exposure on the outer leaflet of cellular membrane**.** In the context of SARS-CoV-2 infection, the intracellular conditions required for TMEM16F scramblase activation, such as cytoplasmic Ca^2+^ elevation, also necessary for PtdSer translocation, may be triggered by viral infection itself and ACE2 downmodulation/Ang II increase/AT1R axis activation. Finally, the PtdSer-mediated TF activation would lead to the formation of thrombi and activation of extrinsic pathway of coagulation by association with factor VII (Fig. 2b) [102, 103].

In summary, based on the main pathophysiological mechanisms involved in PtdSer exposure, it is plausible to think that PtdSer translocation to the outer leaflet of the plasma membrane of the infected alveolar and endothelial cells may be one of the underlying mechanisms in the inflammation and coagulation abnormalities of COVID-19.

## Concluding remarks

SARS-CoV-2 strategies to enhance viral infectivity, along with COVID-19 clinical complications, such as severe acute inflammation and coagulation abnormalities (DIC and the thrombi formation), have resulted in significant obstacles in the development of effective therapies in this pandemic. Therefore, understanding the pathogenic mechanisms underlying the clinical manifestations of COVID-19 has become a priority in the development of new anti-COVID-19 approaches.

The occurrence of these comorbidities could be related to the following main mechanisms: (1) activation of endothelium cells by pro-inflammatory cytokines, known as the “cytokines storm"; (2) viral infection-mediated endothelial cells damage and extrinsic pathway activation, through inducing TF expression; (3) decrease plasma activity of ADAMTS 13 metalloproteinase, with subsequent accumulation of vWF multimers in plasma and formation of platelet microaggregates in the circulation; (4) and RAS unbalancing mediated Ang II high plasma levels following by thrombin activation (Fig. 1).

In this context, the exposure of PtdSer on the outer side of the cell membrane, in cells infected by SARS-CoV-2, emerges as another possible mechanism involved in the genesis of coagulation cascade activation by inducing TF activity. Moreover, the PtdSer exposure on the outer lipid bilayer may contribute to acute inflammation by increasing the sheddase activity of ADAM17, and, consequently, the ACE2, TNF-α, EGFR and IL-6R release, along with other inflammatory molecules leading to acute inflammation (Fig. 2a). Thus, the pathophysiological concepts introduced in this review support the possible participation of PtdSer exposure on the outer side of the SARS-CoV-2 infected cells membrane in the pathophysiology of Covid-19, and open a new avenue of research in the identification of new therapeutic targets against COVID-19.

## Data Availability

Not applicable.

## Abbreviations

ACE2: Angiotensin-converting enzyme 2
TMPRSS2: Type II transmembrane serine protease
RAS: Renin-angiotensin system
ADAM-17: Disintegrin and metalloproteinase domain 17
IL-6R: Interleukin-6 receptor
sACE2: ACE2 soluble form
COPD: Chronic obstructive pulmonary disease
ARDS: Acute respiratory distress syndrome
DIC: Disseminated intravascular coagulation
vWF: Von Willebrand factor
NETs: Neuthrophil extracellular traps
HIF: Hypoxia-inducible factor
TF: Tissue factor
TFPI: Tissue factor pathway inhibitor
PT: Prothrombin time extension
Xkr8: Caspase-dependent Xkr8
LPS: Cytosolic lipopolysaccharide
TAT: Thrombin-antithrombin complex
TMEM16F/anoctamin 6: Transmembrane 16F lipid-scramblase protein
FGF7: Fibroblast growth factor 7
